# Stability Analysis for Nonlinear Impulsive Control System with Uncertainty Factors

**DOI:** 10.1155/2020/8818794

**Published:** 2020-11-21

**Authors:** Zemin Ren, Shiping Wen, Qingyu Li, Yuming Feng, Ning Tang

**Affiliations:** ^1^School of Mathematics, Physics and Data Science, Chongqing University of Science and Technology, Chongqing 401331, China; ^2^Australian AI Institute, University of Technology Sydney, Ultimo, NSW 2007, Australia; ^3^Key Laboratory of Intelligent Information Processing and Control, Chongqing Three Gorges University, Wanzhou, Chongqing 404100, China

## Abstract

Considering the limitation of machine and technology, we study the stability for nonlinear impulsive control system with some uncertainty factors, such as the bounded gain error and the parameter uncertainty. A new sufficient condition for this system is established based on the generalized Cauchy–Schwarz inequality in this paper. Compared with some existing results, the proposed method is more practically applicable. The effectiveness of the proposed method is shown by a numerical example.

## 1. Introduction

Impulse control is based on impulsive differential equation and has many applications [1–6], such as digital communication system, artificial intelligence, and financial sector. In comparison with other methods, impulse control is more efficient in dealing with the stability of complex systems. The stability is an important property of the impulsive control system. Mathematically, its goal is to stabilize an unstable system by proper impulse. Up to now, a wide variety of achievements of impulse control theory have been developed in the literature [7–13].

Generally, there are at least one “impulsively” changeable state variable appearing in a plant *P*, which could be described as following control system:

(1) $$\begin{array}{c} \left\{\begin{array}{c} \dot{x}\left(t\right)=Ax+\phi \left(x\right),t\neq \tau_{k},\\ \Delta x=U\left(k,x\right),t=\tau_{k},k=1,2,\ldots ,\\ x\left(t_{0}\right)=x_{0}. \end{array}\right. \end{array}$$

Here, *x* ∈ *ℝ*^*n*^ denotes the state variable and *U*(*k*, *x*) the impulse control law. We assume that the control instance satisfies

(2) $$\begin{array}{c} t_{0}<\tau_{1}<\cdots \cdots <\tau_{k}<\tau_{k+1}\cdots ,\\ \lim_{k⟶\infty }\tau_{k}=\infty . \end{array}$$

A continuous nonlinear function *ϕ*(*x*) : *ℝ*^*n*^⟶*ℝ*^*n*^ stratifies *ϕ*(*t*, 0) = 0 and ‖*ϕ*(*x*)‖ ≤ *L*‖*x*‖, where *L* is a Lipschitz constant. Many researchers have paid more attention on control system (1) and achieved many sufficient conditions for the stability of these systems [14–20]. Feng et al. consider single state-jumps impulsive systems with periodically time windows and give stability criteria for the new model [21]. To make the nonlinear impulse control system more reasonable, parameter uncertainty and bounded gain error are introduced into the corresponding impulsive differential equations [22–25]. Considering the limitation of machine and technology, Ma et al. investigate stabilization of impulse control systems with gain error and obtain a sufficient criterion for global exponential stability [26]. Zou et al. study impulsive systems with bounded gain error and form a sufficient criterion for the stability [27].

Cauchy–Schwarz inequality is an important tool to study nonlinear systems [28–31]. Recently, Peng et al. generalize the Cauchy–Schwarz inequality, which is used to deduce asymptotic stability for a class of nonlinear control systems [30]. Under the assumption *U*(*k*, *x*) = *BCx*, they study the after nonlinear system:

(3) $$\begin{array}{c} \left\{\begin{array}{c} \dot{x}\left(t\right)=Ax+\phi \left(x\right),t\neq \tau_{k},\\ \Delta x=BCx,t=\tau_{k},k=1,2,\ldots ,\\ x\left(t_{0}\right)=x_{0}, \end{array}\right. \end{array}$$

where *B* and *C* are constant matrixes. Based on the generalized Cauchy–Schwarz inequality, we consider a class of nonlinear impulsive control systems with the parameter uncertainty, which can be written as follows:

(4) $$\begin{array}{c} \left\{\begin{array}{c} \dot{x}\left(t\right)=\left(A+\Delta A\right)x+\phi \left(x\right),t\neq \tau_{k},\\ \Delta x=BCx,t=\tau_{k},k=1,2,\ldots ,\\ x\left(t_{0}\right)=x_{0}. \end{array}\right. \end{array}$$

Generally, one can express the parameter uncertainty as Δ*A* = *GF*(*t*)*H* with *F*^*T*^(*t*)*F*(*t*) ≤ *I*. Here, matrixes *G* and *H* are given with appropriate dimensions. In this paper, we will find some conditions for the stability of system (4). We organize the paper as follows. In Section 2, we briefly introduce some related lemmas. Then, we show sufficient conditions in Section 3. The simulation experiment is shown in Section 4, and conclusion is listed in Section 4.

## 2. Related Lemmas

First of all, we introduce some lemmas to be used later. Throughout this paper, *λ*_max_and*λ*_min_ are denoted as the largest eigenvalue and the smallest eigenvalue, respectively. ‖*·*‖ is denoted as the Euclidian norm of matric or vector.

**Lemma 1 (see [30]).**

Suppose that *P* is positive definite. If *x*, *y* ∈ *R*^*n*^ satisfy |*x*^*T*^*y*| ≤ *σ*(*x*^*T*^*x*)(*y*^*T*^*y*) for a certain *σ* ∈ [0,1], then

(5) $$\begin{array}{c} {\left(x^{T}Py\right)}^{2}\leq {\left(\frac{\lambda_{\max}\left(P\right)-g\left(\sqrt{\sigma }\right)\lambda_{\min}\left(P\right)}{\lambda_{\max}\left(P\right)+g\left(\sqrt{\sigma }\right)\lambda_{\min}\left(P\right)}\right)}^{2}\left(x^{T}Px\right)\left(y^{T}Py\right), \end{array}$$

where *g*(*σ*) = (1 − *σ*/1 + *σ*).

**Lemma 2 .**

(see [27]). Suppose that *Q* is symmetric and positive definite; then, for any *A*, *B* ∈ *R*^*n*×*n*^ and *μ* > 0,

(6) $$\begin{array}{c} A^{T}QB+B^{T}QA\leq \mu A^{T}QA+\frac{1}{\mu }B^{T}QB. \end{array}$$

**Lemma 3 .**

(see [32]). Suppose that *H* is a real symmetric matrix; then,

(7) $$\begin{array}{c} \lambda_{\min}\left(H\right)x^{T}x\leq x^{T}Hx\leq \lambda_{\max}\left(H\right)x^{T}x. \end{array}$$

## 3. The Proposed Results

We give the main results in this section. Specifically, we will analyze the stabilization of impulsive control system (4) with bounded gain error and parameter uncertainty and then list some sufficient conditions which assure the origin of the related systems is asymptotically stable.

**Theorem 1 .**

Suppose *P* ∈ *ℝ*^*n*×*n*^ be a symmetric and positive definite matrix, *λ*_1_ = *λ*_min_(*P*), *λ*_2_ = *λ*_max_(*P*), *I* be the identity matrix, *λ*_3_ be the largest eigenvalue of *P*^−1^(*PA* + *A*^*T*^*P*), and *λ*_4_ be the largest eigenvalue of the matrix *P*^−1^(*I* + *BC*)^*T*^*P*(*I* + *BC*). If

(8) $$\begin{array}{c} \left|x^{T}\left(t\right)\phi \left(x\left(t\right)\right)\right|\leq \sigma \left(x^{T}\left(t\right)x\left(t\right)\right)\left(\phi {\left(x\left(t\right)\right)}^{T}\phi \left(x\left(t\right)\right)\right), \end{array}$$

for a certain *σ* ∈ [0,1] and

(9) $$\begin{array}{c} \left(\lambda_{3}+2\sqrt{\left(\frac{\lambda_{2}\lambda_{\max}\left(G^{T}G\right)\lambda_{\max}\left(H^{T}H\right)}{\lambda_{1}}\right)}+2L\frac{\lambda_{2}-g\left(\sqrt{\sigma }\right)\lambda_{1}}{\lambda_{2}+g\left(\sqrt{\sigma }\right)\lambda_{1}}\sqrt{\frac{\lambda_{2}}{\lambda_{1}}}\right)\left(\tau_{k+1}-\tau_{k}\right)\leq -\ln\left(\gamma \lambda_{4}\right), \end{array}$$

where

(10) $$\begin{array}{c} g\left(\sigma \right)=\frac{1-\sigma }{1+\sigma },\gamma >1. \end{array}$$

then, we obtain that the origin of impulsive control system (4) is asymptotically stable.

**Proof**

We choose the Lyapunov function as follows:

(11) $$\begin{array}{c} V\left(x\left(t\right)\right)=x^{T}\left(t\right)Px\left(t\right). \end{array}$$

When *t* ≠ *τ*_*k*_, we obtain Dini's derivative of *V*(*x*(*t*)) for impulsive control system (4) as follows:

(12) $$\begin{array}{c} D^{+}V\left(x\left(t\right)\right)=2x^{T}\left(t\right)P\left(\left(A+\Delta A\right)x\left(t\right)+\phi \left(x\left(t\right)\right)\right),\\ =2x^{T}\left(t\right)PAx\left(t\right)+2x^{T}\left(t\right)P\Delta Ax\left(t\right)+2x^{T}\left(t\right)P\phi \left(x\left(t\right)\right). \end{array}$$

Next, we will calculate the three parts of the above formula (12), respectively. The matrices *P*^−1^(*PA* + *A*^*T*^*P*) and *P*^−0.5^(*PA* + *A*^*T*^*P*)*P*^−0.5^ have the same eigenvalues. By Lemma 3, we have

(13) $$\begin{array}{c} 2x^{T}\left(t\right)PAx\left(t\right)=x^{T}\left(t\right)\left(PA+A^{T}P\right)x\left(t\right),\\ =\left(x^{T}\left(t\right)P^{0.5}\right)\left(P^{-0.5}\left(PA+A^{T}P\right)P^{-0.5}\right)\left(P^{0.5}x\left(t\right)\right),\\ \leq \lambda_{3}\left(x^{T}\left(t\right)P^{0.5}\right)\left(P^{0.5}x\left(t\right)\right),\\ =\lambda_{3}V\left(x\left(t\right)\right). \end{array}$$

According to the Cauchy–Schwarz inequality, we obtain

(14) $$\begin{array}{c} x^{T}\left(t\right)P\Delta Ax\left(t\right)\leq \sqrt{\left(x^{T}\left(t\right)P^{2}x\left(t\right)\right)\left(x^{T}\left(t\right)\Delta A^{T}\Delta Ax\left(t\right)\right)}. \end{array}$$

Since parameter uncertainty Δ*A* = *GF*(*t*)*H* and *F*^*T*^(*t*)*F*(*t*) ≤ *I*, inequality (14) can be rewritten as

(15) $$\begin{array}{c} 2x^{T}\left(t\right)P\Delta Ax\left(t\right)\leq 2\sqrt{\left(x^{T}\left(t\right)P^{2}x\left(t\right)\right)\left(x^{T}\left(t\right)H^{T}F^{T}\left(t\right)G^{T}GF\left(t\right)Hx\left(t\right)\right)},\\ \leq 2\sqrt{\left(\left(x^{T}\left(t\right)P^{1/2}\right)P\left(P^{1/2}x\left(t\right)\right)\right)\left(x^{T}\left(t\right)H^{T}F^{T}\left(t\right)G^{T}GF\left(t\right)Hx\left(t\right)\right)},\\ \leq 2\sqrt{\left(\lambda_{2}V\left(\left(x\left(t\right)\right)\right)\right)\left(\lambda_{\max}\left(G^{T}G\right)x^{T}\left(t\right)H^{T}IHx\left(t\right)\right)},\\ \leq 2\sqrt{\left(\lambda_{2}V\left(x\left(t\right)\right)\right)\left(\lambda_{\max}\left(G^{T}G\right)\lambda_{\max}\left(H^{T}H\right)x^{T}\left(t\right)x\left(t\right)\right)},\\ =2\sqrt{\left(\lambda_{2}V\left.\left(x\left(t\right)\right)\right)|\left(\lambda_{\max}\left(G^{T}G\right)\lambda_{\max}\left(H^{T}H\right)\left(x^{T}\left(t\right)\right.P^{1/2}\right)P^{-1}\left(P^{\left(1/2\right)}x\left(t\right)\right)\right)},\\ \leq 2\sqrt{\left(\frac{\lambda_{2}\lambda_{\max}\left(G^{T}G\right)\lambda_{\max}\left(H^{T}H\right)}{\lambda_{1}}\right)}V\left(x\left(t\right)\right). \end{array}$$

According to Lemma 1, we obtain

(16) $$\begin{array}{c} 2x^{T}\left(t\right)P\phi \left(x\left(t\right)\right)\leq 2L\frac{\lambda_{2}-g\left(\sqrt{\sigma }\right)\lambda_{1}}{\lambda_{2}+g\left(\sqrt{\sigma }\right)\lambda_{1}}\sqrt{\left(x^{T}\left(t\right)Px\left(t\right)\right)\left(\phi {\left(x\left(t\right)\right)}^{T}P\phi \left(x\left(t\right)\right)\right)},\\ \leq 2L\frac{\lambda_{2}-g\left(\sqrt{\sigma }\right)\lambda_{1}}{\lambda_{2}+g\left(\sqrt{\sigma }\right)\lambda_{1}}\sqrt{\lambda_{2}\left(x^{T}\left(t\right)Px\left(t\right)\right)\left(\phi {\left(x\left(t\right)\right)}^{T}\phi \left(x\left(t\right)\right)\right).} \end{array}$$

Since ‖*ϕ*(*x*)‖ ≤ *L*‖*x*‖, inequality (16) can be obtained as follows:

(17) $$\begin{array}{c} 2x^{T}\left(t\right)P\phi \left(x\left(t\right)\right)\leq 2L\frac{\lambda_{2}-g\left(\sqrt{\sigma }\right)\lambda_{1}}{\lambda_{2}+g\left(\sqrt{\sigma }\right)\lambda_{1}}\sqrt{\lambda_{2}\left(x^{T}\left(t\right)Px\left(t\right)\right)\left(x{\left(t\right)}^{T}x\left(t\right)\right)},\\ \leq 2L\frac{\lambda_{2}-g\left(\sqrt{\sigma }\right)\lambda_{1}}{\lambda_{2}+g\left(\sqrt{\sigma }\right)\lambda_{1}}\sqrt{\lambda_{2}\left(x^{T}\left(t\right)Px\left(t\right)\right)\left(x{\left(t\right)}^{T}x\left(t\right)\right)},\\ \leq 2L\frac{\lambda_{2}-g\left(\sqrt{\sigma }\right)\lambda_{1}}{\lambda_{2}+g\left(\sqrt{\sigma }\right)\lambda_{1}}\sqrt{\left(\frac{\lambda_{2}}{\lambda_{1}}\right)\left(x^{T}\left(t\right)Px\left(t\right)\right)\left(x{\left(t\right)}^{T}Px\left(t\right)\right)},\\ =2L\frac{\lambda_{2}-g\left(\sqrt{\sigma }\right)\lambda_{1}}{\lambda_{2}+g\left(\sqrt{\sigma }\right)\lambda_{1}}\sqrt{\frac{\lambda_{2}}{\lambda_{1}}}V\left(x\left(t\right)\right). \end{array}$$

Combining inequalities (13), (15), and (17), we obtain

(18) $$\begin{array}{c} D^{+}V\left(x\left(t\right)\right)\leq \left(\lambda_{3}+2\sqrt{\left(\frac{\lambda_{2}\lambda_{\max}\left(G^{T}G\right)\lambda_{\max}\left(H^{T}H\right)}{\lambda_{1}}\right)}+2L\frac{\lambda_{2}-g\left(\sqrt{\sigma }\right)\lambda_{1}}{\lambda_{2}+g\left(\sqrt{\sigma }\right)\lambda_{1}}\sqrt{\frac{\lambda_{2}}{\lambda_{1}}}\right)V\left(x\left(t\right)\right). \end{array}$$

When *t* = *τ*_*k*_, we compute the value of *V* as follows:

(19) $$\begin{array}{c} {V\left(x\left(t\right)+BCx\left(t\right)\right)|}_{t=\tau_{k}}={{\left(x\left(t\right)+BCx\left(t\right)\right)}^{T}P\left(x\left(t\right)+BCx\left(t\right)\right)|}_{t=\tau_{k}},\\ ={x{\left(t\right)}^{T}{\left(I+BC\right)}^{T}P\left(I+BC\right)x\left(t\right)|}_{t=\tau_{k}},\\ ={\left(x^{T}\left(t\right)P^{0.5}\right)\left(P^{-0.5}{\left(I+BC\right)}^{T}P\left(I+BC\right)P^{-0.5}\right)\left(P^{0.5}x\left(t\right)\right)|}_{t=\tau_{k}}. \end{array}$$

It is known that the matrix *P*^−0.5^(*I* + *BC*)^*T*^*P*(*I* + *BC*)*P*^−0.5^ has the same eigenvalues with the matrix *P*^−1^(*I* + *BC*)^*T*^*P*(*I* + *BC*). Thus, it follows from (19) that

(20) $$\begin{array}{c} {V\left(x\left(t\right)+BCx\left(t\right)\right)|}_{t=\tau_{k}}\leq {\lambda_{4}\left(x^{T}\left(t\right)P^{0.5}\right)\left(P^{0.5}x\left(t\right)\right)|}_{t=\tau_{k}},\\ ={\lambda_{4}V\left(x\left(t\right)\right)|}_{t=\tau_{k}}. \end{array}$$

Now, we analyze the following comparison system:

(21) $$\begin{array}{c} \dot{\omega }=\left(\lambda_{3}+2\sqrt{\left(\frac{\lambda_{2}\lambda_{\max}\left(G^{T}G\right)\lambda_{\max}\left(H^{T}H\right)}{\lambda_{1}}\right)}+2L\frac{\lambda_{2}-g\left(\sqrt{\sigma }\right)\lambda_{1}}{\lambda_{2}+g\left(\sqrt{\sigma }\right)\lambda_{1}}\sqrt{\frac{\lambda_{2}}{\lambda_{1}}}\right)\omega \left(t\right),t\neq \tau_{k},\\ \omega \left(\tau_{k}^{+}\right)=\lambda_{4}\omega \left(\tau_{k}\right),\\ \omega \left(\tau_{0}^{+}\right)=\omega_{0}\geq 0. \end{array}$$

According to the related conclusion (see Theorem 3 in [29]), we obtain that if

(22) $$\begin{array}{c} \int_{\tau_{k}}^{\tau_{k+1}}\left(\lambda_{3}+2\sqrt{\left(\frac{\lambda_{2}\lambda_{\max}\left(G^{T}G\right)\lambda_{\max}\left(H^{T}H\right)}{\lambda_{1}}\right)}+2L\frac{\lambda_{2}-g\left(\sqrt{\sigma }\right)\lambda_{1}}{\lambda_{2}+g\left(\sqrt{\sigma }\right)\lambda_{1}}\sqrt{\frac{\lambda_{2}}{\lambda_{1}}}\right)\text{d}t+\ln\left(\gamma \lambda_{4}\right)\leq 0,\gamma >1. \end{array}$$

The origin of impulsive control system (4) is asymptotically stable.

**Remark 1 .**

If the parameter uncertainty Δ*A* = 0, the condition of (9) became the result of Theorem 3.1 in reference [30]. Thus, the proposed method is a generalization of Peng's method.

In many practical applications, it is inevitable to put impulses with errors due to the limitation of machine and technology. So, we integrate the bounded gain error into the impulsive system (4). For simplicity, let *D* = *BC*. We rewrite the corresponding system as

(23) $$\begin{array}{c} \left\{\begin{array}{c} x\left(t\right)=\left(A+\Delta A\right)x\left(t\right)+\phi \left(x\left(t\right)\right),t\neq \tau_{k},\\ \Delta x\left(t\right)=\left(D+\Delta D\right)x\left(t\right),t=\tau_{k},k=1,2,\ldots ,\\ x\left(t_{0}\right)=x_{0}, \end{array}\right. \end{array}$$

where Δ*D* denotes the bounded gain error and has the following form: Δ*D* = *mF*(*t*)*D* with *m* > 0 and *F*^*T*^(*t*)*F*(*t*) ≤ *I*. It is easy to obtain a similar analysis from Theorem 1.

**Theorem 2 .**

Let *P* ∈ *ℝ*^*n*×*n*^ be a symmetric and positive definite matrix, *λ*_1_ = *λ*_min_(*P*), *λ*_2_ = *λ*_max_(*P*), *I* be the identity matrix, and *λ*_3_ be the largest eigenvalue of *P*^−1^(*PA* + *A*^*T*^*P*). If

(24) $$\begin{array}{c} \left|x^{T}\left(t\right)\phi \left(x\left(t\right)\right)\right|\leq \sigma \left(x^{T}\left(t\right)x\left(t\right)\right)\left(\phi {\left(x\left(t\right)\right)}^{T}\phi \left(x\left(t\right)\right)\right). \end{array}$$

for a certain *σ* ∈ [0,1] and

(25) $$\begin{array}{c} \left(\lambda_{3}+2\sqrt{\left(\frac{\lambda_{2}\lambda_{\max}\left(G^{T}G\right)\lambda_{\max}\left(H^{T}H\right)}{\lambda_{1}}\right)}+2L\frac{\lambda_{2}-g\left(\sqrt{\sigma }\right)\lambda_{1}}{\lambda_{2}+g\left(\sqrt{\sigma }\right)\lambda_{1}}\sqrt{\frac{\lambda_{2}}{\lambda_{1}}}\right)\left(\tau_{k+1}-\tau_{k}\right)\leq -\ln\left(\gamma \lambda_{4}\right), \end{array}$$

where

(26) $$\begin{array}{c} \lambda_{4}=\frac{\lambda_{2}}{\lambda_{1}}\left(\left(1+\mu \right)\lambda_{\max}\left({\left(I+D\right)}^{T}\left(I+D\right)\right)+\left(1+\frac{1}{\mu }\right)m^{2}\lambda_{\max}\left(D^{T}D\right)\right), \end{array}$$

(27) $$\begin{array}{c} g\left(\sigma \right)=\frac{1-\sigma }{1+\sigma },\gamma >1. \end{array}$$

Then, the origin of impulsive control system (23) is asymptotically stable.

**Proof**

We choose the following Lyapunov function as follows:

(28) $$\begin{array}{c} V\left(x\left(t\right)\right)=x^{T}\left(t\right)Px\left(t\right). \end{array}$$

According to inequality (18), Dini's derivative of *V*(*x*(*t*)) for impulsive control system (23) is acquired as follows:

(29) $$\begin{array}{c} D^{+}V\left(x\left(t\right)\right)\leq \left(\lambda_{3}+2\sqrt{\left(\frac{\lambda_{2}\lambda_{\max}\left(G^{T}G\right)\lambda_{\max}\left(H^{T}H\right)}{\lambda_{1}}\right)}+2L\frac{\lambda_{2}-g\left(\sqrt{\sigma }\right)\lambda_{1}}{\lambda_{2}+g\left(\sqrt{\sigma }\right)\lambda_{1}}\sqrt{\frac{\lambda_{2}}{\lambda_{1}}}\right)V\left(x\left(t\right)\right). \end{array}$$

Then, we just need to compute *V*(*x*(*t*) + (*D* + Δ*D*)*x*(*t*))*|*_*t*=*τ*_*k*__.

We perform some calculations on *V*(*x*(*t*) + (*D* + Δ*D*)*x*(*t*))*|*_*t*=*τ*_*k*__and obtain

(30) $$\begin{array}{c} {V\left(x\left(t\right)+\left(D+\Delta D\right)x\left(t\right)\right)|}_{t=\tau_{k}}={{\left(x\left(t\right)+\left(D+\Delta D\right)x\left(t\right)\right)}^{T}P\left(x\left(t\right)+\left(D+\Delta D\right)x\left(t\right)\right)|}_{t=\tau_{k}},\\ ={x{\left(t\right)}^{T}{\left(\left(I+D\right)+\Delta D\right)}^{T}P\left(\left(I+D\right)+\Delta D\right)x\left(t\right)|}_{t=\tau_{k}},\\ \leq {\lambda_{2}x{\left(t\right)}^{T}{\left(\left(I+D\right)+\Delta D\right)}^{T}\left(\left(I+D\right)+\Delta D\right)x\left(t\right)|}_{t=\tau_{k}},\\ \leq {\lambda_{2}x{\left(t\right)}^{T}\left({\left(I+D\right)}^{T}\left(I+D\right)+{\left(I+D\right)}^{T}\Delta D+\Delta D^{T}\left(I+D\right)+\Delta D^{T}\Delta D\right)x\left(t\right)|}_{t=\tau_{k}}. \end{array}$$

By using Lemma 2 and Δ*D* = *mF*(*t*)*D*, we rewrite inequality (30) as

(31) $$\begin{array}{c} {V\left(x\left(t\right)+\left(D+\Delta D\right)x\left(t\right)\right)|}_{t=\tau_{k}}\leq {\lambda_{2}x^{T}\left(t\right)\left({\left(I+D\right)}^{T}\left(I+D\right)+{\left(I+D\right)}^{T}\Delta D+\Delta D^{T}\Delta D+\Delta D^{T}\left(I+D\right)\right)x\left(t\right)|}_{t=\tau_{k}},\\ \leq {\lambda_{2}x^{T}\left(t\right)\left(\left(1+\mu \right){\left(I+D\right)}^{T}\left(I+D\right)+\left(1+\frac{1}{\mu }\right)\Delta D^{T}\Delta D\right)x\left(t\right)|}_{t=\tau_{k}},\\ ={\lambda_{2}x^{T}\left(t\right)\left(\left(1+\mu \right){\left(I+D\right)}^{T}\left(I+D\right)+\left(1+\frac{1}{\mu }\right)m^{2}D^{T}F^{T}\left(t\right)F\left(t\right)D\right)x\left(t\right)|}_{t=\tau_{k}}. \end{array}$$

It follows from (15) that

(32) $$\begin{array}{c} x^{T}\left(t\right)x\left(t\right)=\left(x^{T}\left(t\right)P^{1/2}\right)P^{-1}\left(P^{1/2}x\left(t\right)\right)\leq \frac{V\left(x\left(t\right)\right)}{\lambda_{1}}. \end{array}$$

Combine inequalities (31) and (32) and *F*^*T*^(*t*)*F*(*t*) ≤ *I*, we obtain

(33) $$\begin{array}{c} {V\left(x\left(t\right)+\left(D+\Delta D\right)x\left(t\right)\right)|}_{t=\tau_{k}}={\lambda_{2}x^{T}\left(t\right)\left(\left(1+\mu \right){\left(I+D\right)}^{T}\left(I+D\right)+\left(1+\frac{1}{\mu }\right)m^{2}D^{T}F^{T}\left(t\right)F\left(t\right)D\right)x\left(t\right)|}_{t=\tau_{k}},\\ \leq {\lambda_{2}x^{T}\left(t\right)\left(\left(1+\mu \right){\left(I+D\right)}^{T}\left(I+D\right)+\left(1+\frac{1}{\mu }\right)m^{2}D^{T}D\right)x\left(t\right)|}_{t=\tau_{k}},\\ \leq {\frac{\lambda_{2}}{\lambda_{1}}\left(\left(1+\mu \right)\lambda_{\max}\left({\left(I+D\right)}^{T}\left(I+D\right)\right)+\left(1+\frac{1}{\mu }\right)m^{2}\lambda_{\max}\left(D^{T}D\right)\right)V\left(x\left(t\right)\right)|}_{t=\tau_{k}},\\ ={\lambda_{4}V\left(x\left(t\right)\right)|}_{t=\tau_{k}}. \end{array}$$

Here, we emit the rest analysis process, which is similar to Theorem 1. Thus, from equalities (29) and (33), we obtain that if

(34) $$\begin{array}{c} \left(\lambda_{3}+2\sqrt{\left(\frac{\lambda_{2}\lambda_{\max}\left(G^{T}G\right)\lambda_{\max}\left(H^{T}H\right)}{\lambda_{1}}\right)}+2L\frac{\lambda_{2}-g\left(\sqrt{\sigma }\right)\lambda_{1}}{\lambda_{2}+g\left(\sqrt{\sigma }\right)\lambda_{1}}\sqrt{\frac{\lambda_{2}}{\lambda_{1}}}\right)\left(\tau_{k+1}-\tau_{k}\right)\leq -\ln\left(\gamma \lambda_{4}\right),\\ \lambda_{4}=\frac{\lambda_{2}}{\lambda_{1}}\left(\left(1+\mu \right)\lambda_{\max}\left({\left(I+D\right)}^{T}\left(I+D\right)\right)+\left(1+\frac{1}{\mu }\right)m^{2}\lambda_{\max}\left(D^{T}D\right)\right), \end{array}$$

the origin of impulsive control system (23) is asymptotically stable. This completes the proof.

## 4. A Numerical Example

In this section, we perform the proposed model on a numerical example to display its effectiveness. The example is produced by Qi and Chen [33]. Let *x* = [*x*_1_, *x*_2_, *x*_3_]^*T*^, *ϕ*(*x*) = [*x*_2_*x*_3_, −*x*_1_*x*_3_, *x*_1_*x*_2_]^*T*^, and

(35) $$\begin{array}{c} A=\left[\begin{array}{ccc} -a & a & 0\\ c & -1 & 0\\ 0 & 0 & -b \end{array}\right]. \end{array}$$

The corresponding state equation can be described as

(36) $$\begin{array}{c} \dot{x}=Ax+\phi \left(x\right). \end{array}$$

**Figure 1:**
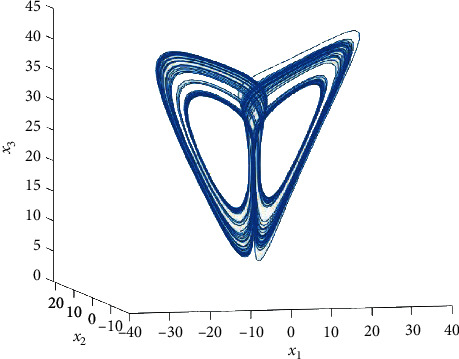
The chaotic phenomenon of system (36) with the initial condition: *x*(0) = [3,5,10]^*T*^.

According to the strategy of [33], some parameters of this system are set as *a* = 35, *b* = (8/3), and *c* = 25. From Figure 1, we can see that system (36) is chaotic for the initial condition: *x*(0) = [3,5,10]^*T*^.

After simple calculation, we obtain that

(37) $$\begin{array}{c} \left\|\phi \left(x\right)\right\|=\sqrt{{\left(x_{2}x_{3}\right)}^{2}+{\left(x_{1}x_{3}\right)}^{2}+{\left(x_{1}x_{2}\right)}^{2}},\\ \leq \max\left\{\left|x_{1}\right|,\left|x_{2}\right|,\left|x_{3}\right|\right\}\sqrt{x_{1}^{2}+x_{2}^{2}+x_{3}^{2}},\\ =\max\left\{\left|x_{1}\right|,\left|x_{2}\right|,\left|x_{3}\right|\right\}\left\|x\right\|. \end{array}$$

From Figure 1, we can intuitively find max{|*x*_1_|, |*x*_2_|, |*x*_3_|} ≤ 45. Combining with inequality (37), the parameter *L* can be set as 45. Since

(38) $$\begin{array}{c} {\left|x^{T}\phi \left(x\right)\right|}^{2}\leq \frac{1}{9}\left(x^{T}x\right)\left(\phi {\left(x\right)}^{T}\phi \left(x\right)\right), \end{array}$$

the parameter *σ* is chosen as *σ* = (1/9). In this section, some matrices are chosen as follows:

(39) $$\begin{array}{c} G=H=\left[\begin{array}{ccc} 0.5 & 0 & 0\\ 0 & 0.5 & 0\\ 0 & 0 & 0.5 \end{array}\right],\\ P=C=\left[\begin{array}{ccc} 1 & 0 & 0\\ 0 & 1 & 0\\ 0 & 0 & 1 \end{array}\right],\\ B=\left[\begin{array}{ccc} -0.5 & -0.01 & 0.02\\ -0.01 & -0.5 & 0\\ 0.02 & 0 & -0.5 \end{array}\right]. \end{array}$$

Thus, the parameter uncertainty can be formed as

(40) $$\begin{array}{c} \Delta A=\left[\begin{array}{ccc} 0.5 & 0 & 0\\ 0 & 0.5 & 0\\ 0 & 0 & 0.5 \end{array}\right]\left[\begin{array}{ccc} 0.1\sin\left(t\right) & 0 & 0\\ 0 & 0.1\sin\left(t\right) & 0\\ 0 & 0 & 0.1\sin\left(t\right) \end{array}\right]\left[\begin{array}{ccc} 0.5 & 0 & 0\\ 0 & 0.5 & 0\\ 0 & 0 & 0.5 \end{array}\right]. \end{array}$$

According to Theorem 1, we calculate *λ*_3_ = 32.9638 and *λ*_4_ = 0.2729. It follows from (8) that

(41) $$\begin{array}{c} \tau_{k+1}-\tau_{k}\leq -\frac{\ln\left(\gamma \lambda_{4}\right)}{63.4638}. \end{array}$$

**Figure 2:**
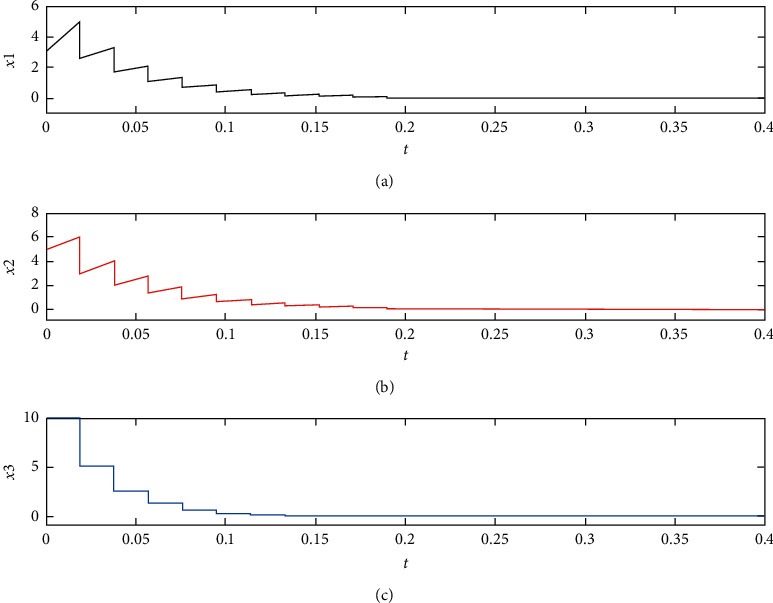
Time response curves for the controlled system (36) with the parameter uncertainty.

If *γ* = 1.1, it yields *τ*_*k*+1_ − *τ*_*k*_ ≤ 0.0190. We choose *τ*_*k*+1_ − *τ*_*k*_ = 0.0190 and show the simulation result in Figure 2. The impulsive control system (36) is asymptotically stable.

Next, we consider the controlled system (36) with the parameter uncertainty and the bounded gain error. The gain error is detailed as Δ*D* = *m*sin(*t*)*D* in this section. We perform some similar calculation on (25) and obtain *λ*_3_ = 32.9638. We choose *μ* = 1 and then obtain *λ*_4_ = 0.5458(1 + *m*^2^) from (26). Let *γ* = 1.1 and *m* = 0.05; then,

(42) $$\begin{array}{c} \tau_{k+1}-\tau_{k}\leq 0.0080. \end{array}$$

**Figure 3:**
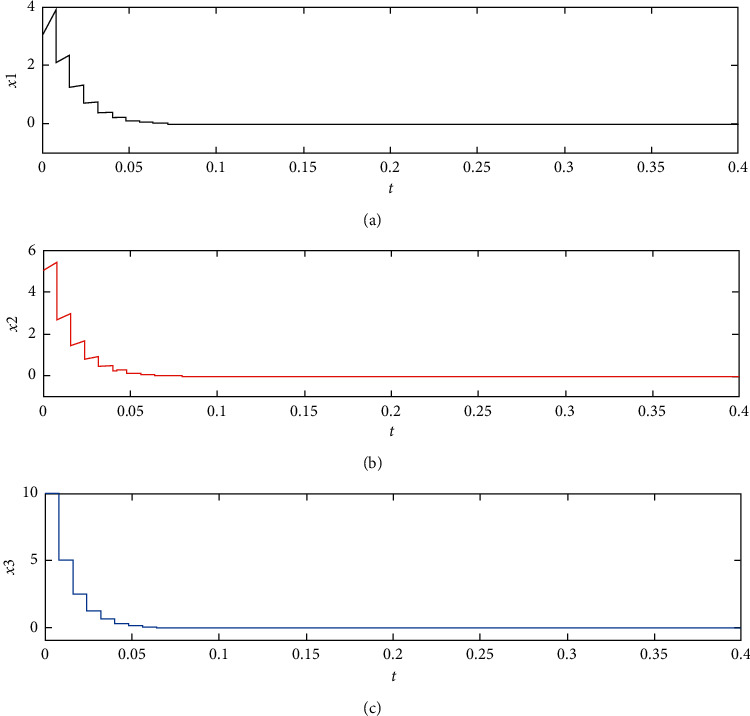
Time response curves for the controlled system (36) with parameter uncertainty and gain error.

Thus, we choose *τ*_*k*+1_ − *τ*_*k*_ = 0.0080 and show the experimental result in Figure 3. From this figure, we can obtain that the impulsive control system (36) is asymptotically stable.

## 5. Conclusion

We study the asymptotic stability of impulsive control systems with some uncertainty factors, such as the bounded gain error and the parameter. The proposed sufficient condition is established based on the generalized Cauchy–Schwarz inequality. We think the proposed issue is more practically applicable than some existing ones.

## Data Availability

No data were used to support this study.
